# Maltine

**Published:** 1879-04

**Authors:** 


MALTINE.—Our market is now supplied with the Maltine preparations furnished by Reed & Carnrick. We have made trial of these preparations and can now safely say, our patients so far as heard from, are well pleased with their effects. Maltine Wine is an especial favorite with some, and I must confess myself among the number.

